# Investigation of the thermophilic mechanism in the genus *Porphyrobacter* by comparative genomic analysis

**DOI:** 10.1186/s12864-018-4789-4

**Published:** 2018-05-23

**Authors:** Lin Xu, Yue-Hong Wu, Peng Zhou, Hong Cheng, Qian Liu, Xue-Wei Xu

**Affiliations:** 1 Key Laboratory of Marine Ecosystem and Biogeochemistry, Second Institute of Oceanography, State Oceanic Administration, 310012 Hangzhou, People’s Republic of China; 2 College of Life Sciences, Zhejiang Sci-Tech University, 310018 Hangzhou, People’s Republic of China; 3 Ocean College, Zhejiang University, 316021 Zhoushan, People’s Republic of China

**Keywords:** *Porphyrobacter*, Comparative genomics, Thermophiles, Adaptational mechanism, Amino acid substitution

## Abstract

**Background:**

Type strains of the genus *Porphyrobacter* belonging to the family *Erythrobacteraceae* and the class *Alphaproteobacteria* have been isolated from various environments, such as swimming pools, lake water and hot springs. *P. cryptus* DSM 12079^T^ and *P*. *tepidarius* DSM 10594^T^ out of all *Erythrobacteraceae* type strains, are two type strains that have been isolated from geothermal environments. Next-generation sequencing (NGS) technology offers a convenient approach for detecting situational types based on protein sequence differences between thermophiles and mesophiles; amino acid substitutions can lead to protein structural changes, improving the thermal stabilities of proteins. Comparative genomic studies have revealed that different thermal types exist in different taxa, and few studies have been focused on the class *Alphaproteobacteria*, especially the family *Erythrobacteraceae*. In this study, eight genomes of *Porphyrobacter* strains were compared to elucidate how *Porphyrobacter* thermophiles developed mechanisms to adapt to thermal environments.

**Results:**

*P. cryptus* DSM 12079^T^ grew optimally at 50 °C, which was higher than the optimal growth temperature of other *Porphyrobacter* type strains. Phylogenomic analysis of the genus *Porphyrobacter* revealed that *P. cryptus* DSM 12079^T^ formed a distinct and independent clade. Comparative genomic studies uncovered that 1405 single-copy genes were shared by *Porphyrobacter* type strains. Alignments of single-copy proteins showed that various types of amino acid substitutions existed between *P. cryptus* DSM 12079^T^ and the other *Porphyrobacter* strains. The primary substitution types were changes from glycine/serine to alanine.

**Conclusions:**

*P. cryptus* DSM 12079^T^ was the sole thermophile within the genus *Porphyrobacter*. Phylogenomic analysis and amino acid frequencies indicated that amino acid substitutions might play an important role in the thermophily of *P. cryptus* DSM 12079^T^. Bioinformatic analysis revealed that major amino acid substitutional types, such as changes from glycine/serine to alanine, increase the frequency of α-helices in proteins, promoting protein thermostability in *P. cryptus* DSM 12079^T^. Hence, comparative genomic analysis broadens our understanding of thermophilic mechanisms in the genus *Porphyrobacter* and may provide a useful insight in the design of thermophilic enzymes for agricultural, industrial and medical applications.

**Electronic supplementary material:**

The online version of this article (10.1186/s12864-018-4789-4) contains supplementary material, which is available to authorized users.

## Background

There are nine species in the genus *Porphyrobacter* at the time of writing [1–9], eight species of which have been validly published (except for *Candidatus* Porphyrobacter meromictius) according to the List of Prokaryotic Names with Standing in Nomenclature [10]. *Porphyrobacter* species live in diverse environments, including hot springs [4, 6], fresh water [2, 7], stadium seats [1], seawater [5, 8, 9], and swimming pools [3]. Among all type strains within the family *Erythrobacteraceae*, *P. cryptus* and *P*. *tepidarius* are the only two species isolated from geothermal environments. Moreover, the genus *Porphyrobacter* has potential applications, such as hydrocarbon degradation [11], algalytic activity [12] and bioleaching [13]. Type strains isolated from hot springs may provide an insight to how these bacteria have adapted to thermal environments. This information may help us design thermophilic enzymes for many important applications.

Thermophiles are microorganisms whose optimum temperature for growth exceeds 45 °C [14]. Thermophiles are widely distributed [14–16] and have been isolated from various geothermal environments, such as artificial hot environments [17–19], marine hydrothermal vents [20–22] and hot springs [23–25]. The thermal stabilities of proteins play an important role in the survival of thermophiles [14, 26]. Amino acid substitutions in protein sequences can improve the thermal stabilities of proteins, which are beneficial changes that help microorganisms adapt to the environment [27–29]. Compared with the proteins of mesophiles, the proteins of thermophiles have a larger fraction of residues in α-helices, enhancing the stability of proteins [30]. Moreover, the proteins of thermophiles often have hydrophobic residues in the protein interior, promoting conformational stability in the inner part of proteins [15, 31]. Therefore, it is worth studying amino acid substitutions in the proteins of thermophiles that exhibit a close phylogenetic relationship to elucidate their adaptational mechanisms to geothermal environments and guide site-directed substitutions in the engineering of industrial enzymes to improve their thermostability.

Next-generation sequencing (NGS) technology generates massive data sets, which offer a convenient approach to studying amino acid substitutions in the protein sequences of microbes. Nishio et al. [32] studied the amino acid substitutions of *Corynebacterium efficiens* with 45 °C as an upper temperature limit for growth (UTLG) by comparing the genomes of C. *efficiens* (UTLG: 40 °C) and *C*. *glutamicum* and pointed out that three substitutions, changing the amino acid from lysine to arginine, serine to alanine or serine to threonine, enhanced the thermostability of proteins. Takami et al. [33] first sequenced the complete genome of a thermophilic *Bacillus*-related species, *Geobacillus kaustophilus* HTA426, with 60 °C as an optimal temperature for growth (OTG) and compared its genome with those of five mesophilic (OTG: 20–45 °C) *Bacillus*-related species, namely, *Bacillus anthracis* Ames, *B. cereus* ATCC 14579, *B*. *halodurans* C-125, *B. subtilis* 168 and *Oceanobacillus iheyensis* HTE831, and reported that arginine, alanine, glycine, proline and valine amino acid substitutions were found at greater than expected frequency in *G*. *kaustophilus* proteins, whereas asparagine, glutamine, serine and threonine were found at lower than expected frequency. However, Singer and Hickey [34] analyzed the genomes of thermophilic *Aquificae* (OTG: 85 °C), *Crenarchaeota* (OGT: 57.5–97.5 °C), *Euryarchaeota* (OTG: 65–95 °C) and *Thermotogae* (OGT: 75 °C) and found that the proportion of glutamic acid, isoleucine, tyrosine and valine increased in thermophilic strains while the proportion of alanine, glutamine, histidine and threonine decreased. Generally, an increase in the content of hydrophobic, charged and aromatic residues and a decrease in the content of polar uncharged residues occurs in thermophile proteins [32–34]. However, the types of amino acid substitutions have been observed to vary in different thermophilic taxa [32–34]. Therefore, studying the amino acid substitution types of various thermophiles, including their genera, will broaden our understanding of their thermophilic mechanisms.

Although some comparative genomic studies concerning amino acid substitutions in thermophile proteins have been performed [32–34], few studies have been focused on the class *Alphaproteobacteria*, especially the family *Erythrobacteraceae*. To interpret the thermophilic mechanisms in this taxon, here, we sequenced the genomes of five *Porphyrobacter* type strains using a NGS platform and performed comparative genomic and statistical analysis among eight genomes of the genus *Porphyrobacter* type strains.

## Results and discussion

Range and optimum growth temperature of type strains belonging to the genus *Porphyrobacter.*

**Fig. 1:**
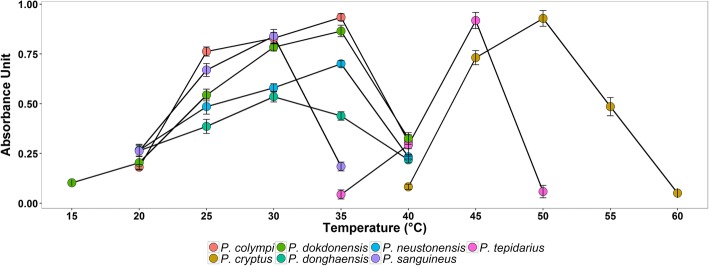
Ranges and optimum growth temperatures for seven *Porphyrobacter* type strains

Except for *P. cryptus* DSM 12079^T^ and *P*. *tepidarius* DSM 10594^T^, other *Porphyrobacter* strains, whose optimal temperature for growth was in the range of 30–35 °C, could not grow at 45 °C (Fig. 1). The optimal growth temperatures for *P. cryptus* DSM 12079^T^ and *P*. *tepidarius* DSM 10594^T^ were 50 and 45 °C, respectively (Fig. 1). Furthermore, the ranges for the growth of *P. cryptus* DSM 12079^T^ and *P*. *tepidarius* DSM 10594^T^ were 40–60 and 35–50 °C, respectively (Fig. 1); the upper limits of these ranges were higher than those of other *Porphyrobacter* species.

Although the optimal temperatures for the growth of *P. cryptus* DSM 12079^T^ and *P*. *tepidarius* DSM 10594^T^ were higher than those of other *Porphyrobacter* strains, *P. cryptus* DSM 12079^T^ was the only thermophile within the genus *Porphyrobacter* based on the definition of a thermophile, which has an optimal temperature that exceeds 45 °C [14]. Notably, *P. cryptus* DSM 12079^T^ could grow above 50 °C, whereas *P*. *tepidarius* DSM 10594^T^ could not.

### Comparative genomic and phylogenomic analyses

The genome size, number of coding sequences (CDSs), and G + C content of the genus *Porphyrobacter* were 2.9–4.3 Mbp, 2839–4042 and 63.6–67.9 mol%, respectively (Table 1). Compared with other *Porphyrobacter* strains, *P. cryptus* DSM 12079^T^ had the smallest genome size and the second lowest number of CDSs (Table 1). However, the *p* values of significant tests on genome size and the number of CDSs between *P. cryptus* DSM 12079^T^ and other *Porphyrobacter* strains were 0.056 and 0.100, respectively, which showed that *P. cryptus* was not significantly different from other *Porphyrobacter* species in terms of genome size and the number of CDSs. Therefore, the small genomic size and reduced number of CDSs were not related to the thermophily of *P. cryptus* DSM 12079^T^.

**Table 1:** Habitats and genomic information of the type strains belonging to the genus *Porphyrobacter*

Type Strain	Habitat	Contigs Number	Total (bp)	N50 Value	G + C Content (mol%)	CDSs Number
*P. colymbi* JCM 18338^T^	Swimming Pool	53	4,309,164	443,561	66.5	4045
*P. cryptus* DSM 12079^T^	Hot Spring	34	2,954,426	268,332	67.8	2852
*P. dokdonensis* DSM 17193^T^	Seawater	13	2,996,446	2,986,864	64.8	2838
*P. donghaensis* DSM 16220^T^	Seawater	11	3,372,281	911,943	66.2	3152
*P. mercurialis* Coronado^T^	Stadium Seat	10	3,482,341	2,089,745	67.3	3154
*P. neustonensis* DSM 9434^T^	Lake	1	3,090,363	3,090,363	65.3	2902
*P. sanguineus* JCM 20691^T^	Seawater	39	3,018,448	462,701	63.6	2884
*P. tepidarius* DSM 10594^T^	Hot Spring	32	3,216,818	372,825	65.9	3104

**Fig. 2:**
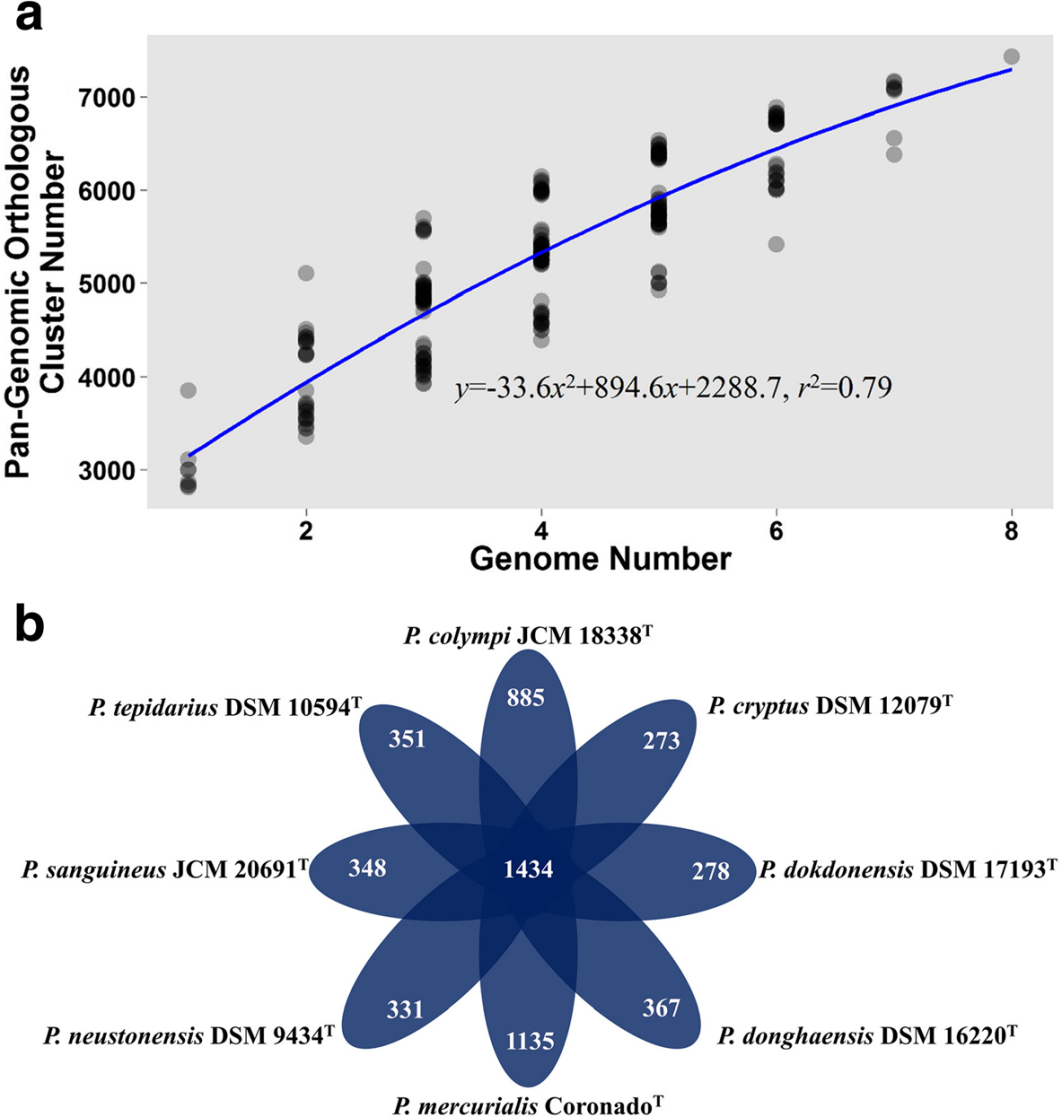
Pan-genomic curve (**a**) showing the relationship between OC number and genome number. Schematic view (**b**) of the core and unique OCs of *Porphyrobacter* species. Central number indicates the shared OCs of the genus *Porphyrobacter*, whereas the ambient number represents the unique OCs harboured by each *Porphyrobacter* species

The number of unique OCs harbored by each *Porphyrobacter* strain varied. *P. mercurialis* Coronado^T^ had the most unique OCs, and *P. cryptus* DSM 12079^T^, being a thermophile, harbored the fewest unique OCs (Fig. 2). It was found that over 80% of the unique OCs held by *P. cryptus* DSM 12079^T^ were shared by the closest relatives belonging to the orders *Rhizobiales*, *Rhodobacterales*, *Rhodospirillales* and *Sphingomonadales*, which do not include typical thermophiles.

**Fig. 3:**
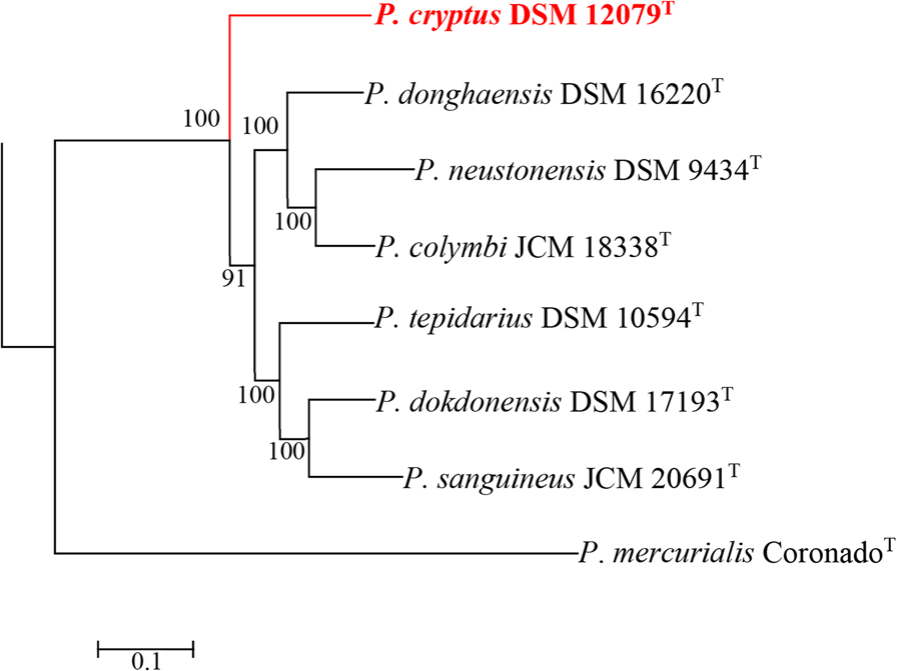
Maximum-likelihood tree based on the contatenatation of 1322 protein sequences showing the phylogenetic relationship of type strains belonging to the genus *Porphyrobacter*. Bootstrap values are based on 100 replicates. Bar, 0.1 substitutions per amino acid position. Red branch shows the clade within *P. cryptus* DSM 12079^T^. *Altererythrobacter epoxidivorans* CGMCC 1.7731^T^ was used as an outgroup (not shown)

Analysis of the genus *Porphyrobacter* showed that their pan-genome was open (Fig. 2), which suggests that the genus *Porphyrobacter* colonizes multiple environments [35], matching the isolation sources of the genus *Porphyrobacter* (Table 1). It was found that 1434 OCs were shared by all eight *Porphyrobacter* strains (Fig. 2). When screening the single-copy shared OCs of eight *Porphyrobacter* strains and *Altererythrobacter epoxidivorans* CGMCC 1.7731^T^, the number of shared OCs decreased to 1322. A maximum-likelihood phylogenetic tree reconstructed based on 1322 shared protein sequences revealed that *P. cryptus* DSM 12079^T^ formed a distinct and independent clade from other *Porphyrobacter* strains (Fig. 3). The phylogenetic distance from other members of the same genus may be the result of amino acid substitutions that have been selected for to allow the organism to adapt to thermal environments.

### Analysis of amino acid substitutions

**Fig. 4:**
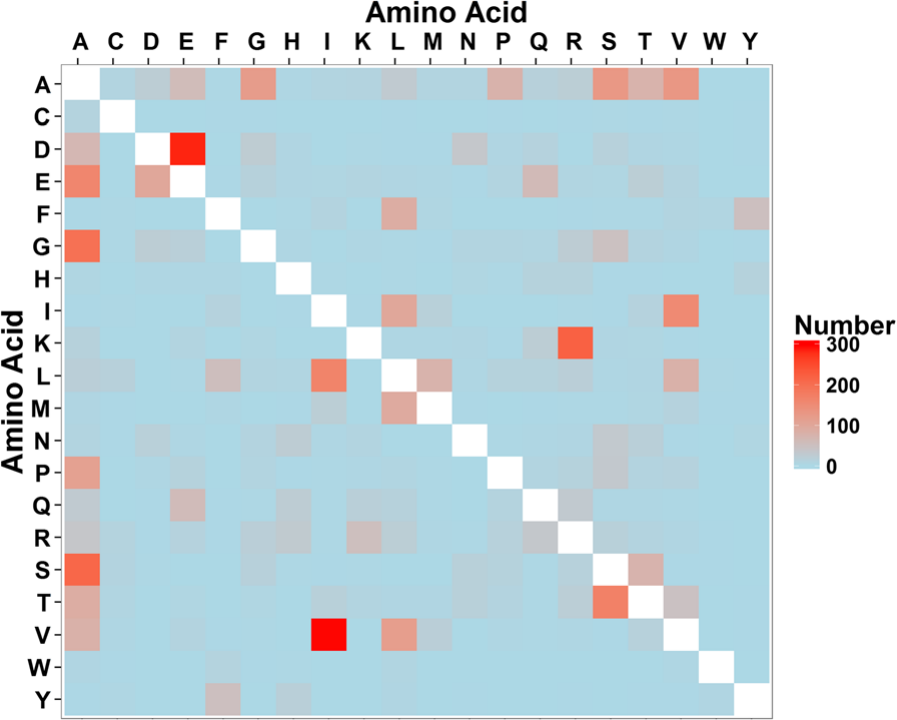
Hot spot map of amino acid variations of *Porphyrobacter cryptus*. Amino acids located on the vertical axis represent substituting amino acids, whereas amino acids lying on the horizontal axis are substituted amino acids. A, alanine; C, cysteine; D, aspartic acid; E, glutamic acid; F, phenylalanine; G, glycine; H, histidine; I, isoleucine; K, lysine; L, lysine; M, methionine; N, asparagine; P, proline; Q, glutamine; R, arginine; S, serine; T, threonine; V, valine; W tryptophan; Y tyrosine

**Fig. 5:**
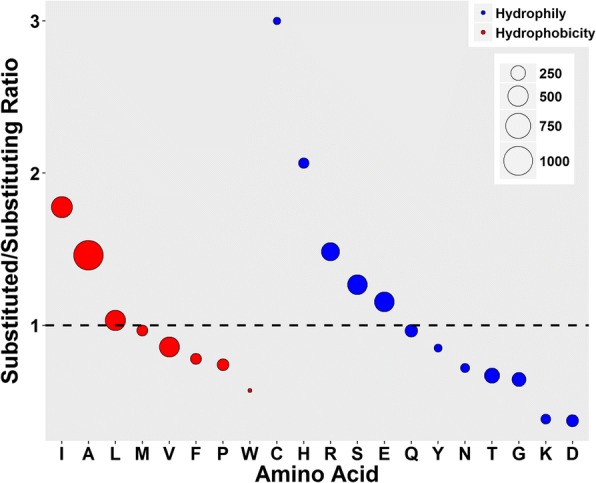
Net substitutional variations of each *Porphyrobacter cryptus* DSM 12079^T^ amino acid based on the alignments of proteins encoded by single-copy genes of the genus *Porphyrobacter*. The dashed line shows the position at which the net variation equals zero. Red indicates hydrophobic amino acids, while blue represents hydrophilic amino acids. A, alanine; C, cysteine; D, aspartic acid; E, glutamic acid; F, phenylalanine; G, glycine; H, histidine; I, isoleucine; K, lysine; L, lysine; M, methionine; N, asparagine; P, proline; Q, glutamine; R, arginine; S, serine; T, threonine; V, valine; W tryptophan; Y tyrosine

There were 274 amino acid substitutions in *P. cryptus* DSM 12079^T^, and the total number that varied amongst all strains was 5698 (Fig. 4, Additional files 1 and 2). Among the whole amino acid substitutions, the major variations (> 3.0%) were changes from valine to isoleucine, asparatic acid to glutamic acid, lysine to arginine, serine to alanine, and glycine to alanine. When compared with proteins from other thermophiles, where the common amino substitutions reported were changes from lysine to arginine and serine to alanine, the number of variations from valine to isoleucine, asparatic acid to glutamic acid, and glycine to alanine were higher here than in other studies [32–34]. Due to bidirectional substitutions, the substituted/substituting ratio analysis indicated that amino acids were most frequently changed to alanine, which resulted in a net substitutional number that was higher for alanine (a ratio above one) than for other amino acids (Fig. 5, Additional file 3).

**Fig. 6:**
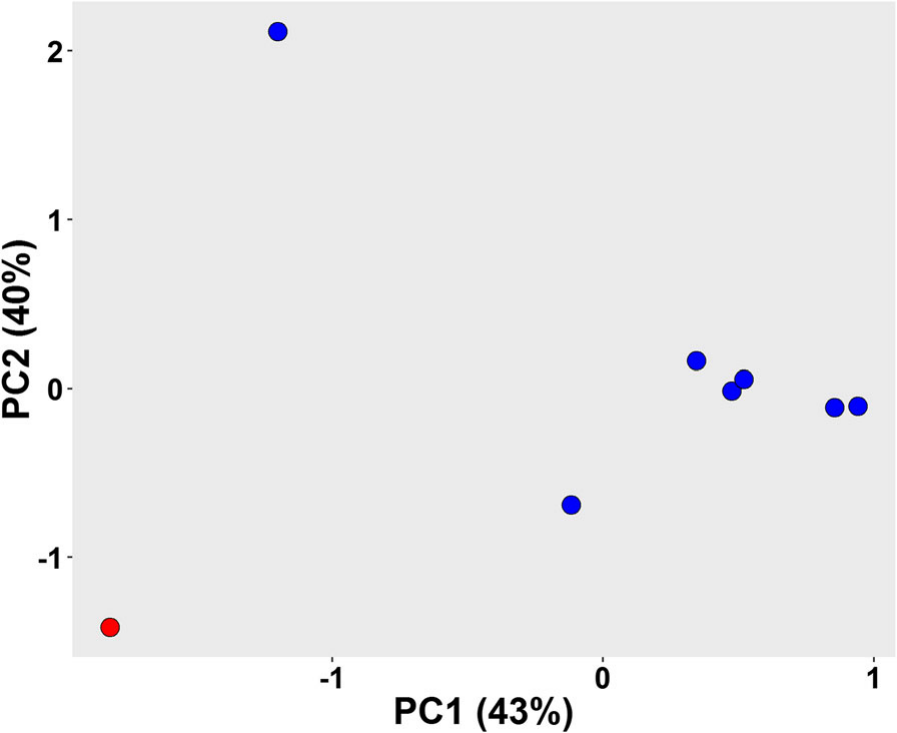
Principal component analysis (PCA) of amino acid frequencies among eight *Porphyrobacter* type strains. PC1 and PC2 account for 43 and 40% of the total variances. Red indicates *P. cryptus* DSM 12079^T^, whereas blue represents seven other *Porphyrobacter* type strains

**Fig. 7:**
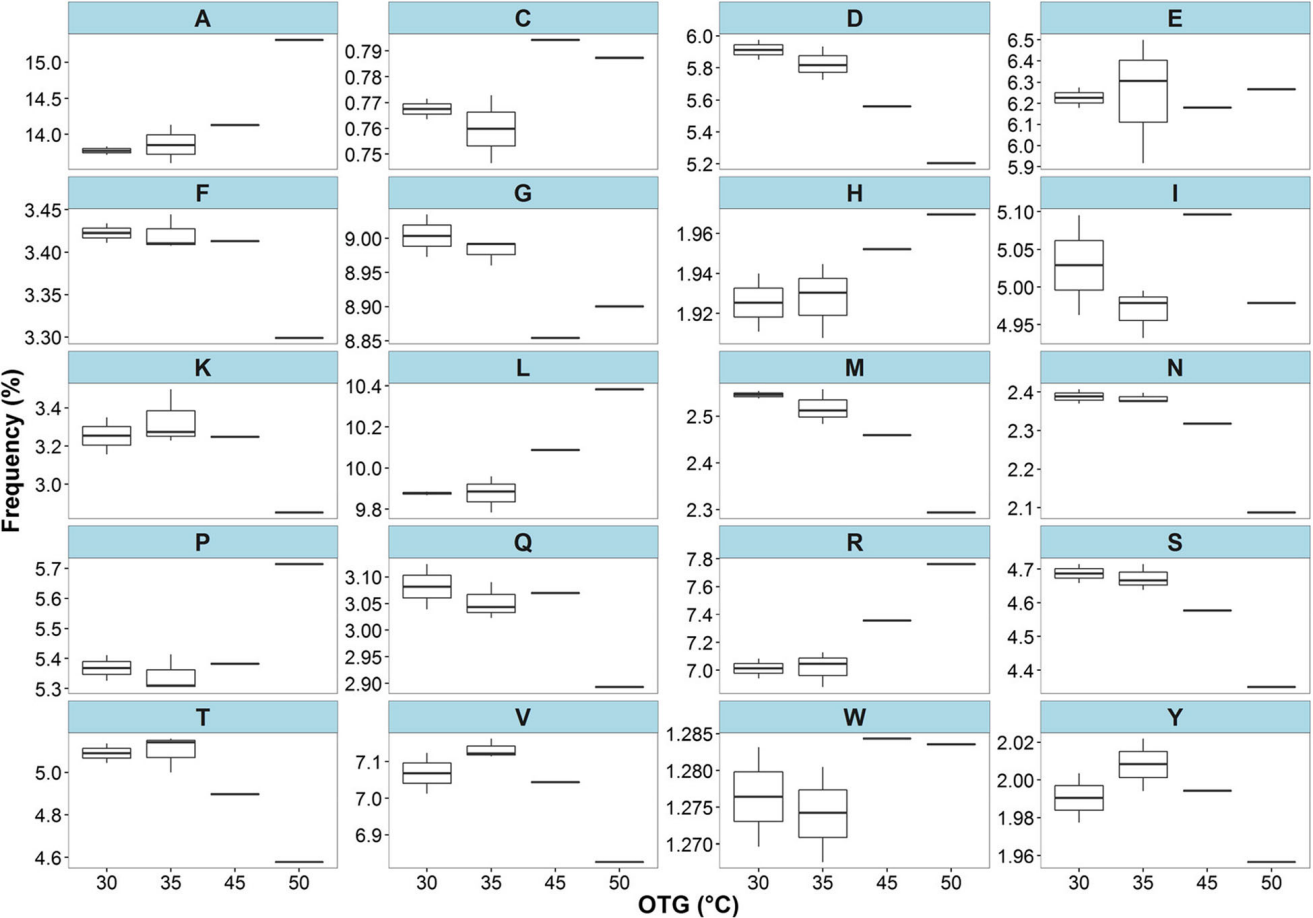
Comparisons of amino acid frequencies among shared OCs in eight *Porphyrobacter* type strains. A, alanine; C, cysteine; D, aspartic acid; E, glutamic acid; F, phenylalanine; G, glycine; H, histidine; I, isoleucine; K, lysine; L, lysine; M, methionine; N, asparagine; P, proline; Q, glutamine; R, arginine; S, serine; T, threonine; V, valine; W tryptophan; Y tyrosine. OTG of *P*. *donghaensis* DSM 16220^T^ and *P. sanguineus* JCM 20691^T^ is 30 °C; OTG of *P*. *colympi* JCM 18338^T^, *P*. *dokdonensis* DSM 17193^T^ and *P*. *neustonensis* DSM 9434^T^ is 35 °C; OTG of *P*. *tepidarius* DSM 10594^T^ is 45 °C; OTG of *P. cryptus* DSM 12079^T^ is 50 °C

In view of amino acid substitutions causing variations in amino acid frequencies, principal component analysis (PCA) of the amino acid frequency revealed that *P. cryptus* DSM 12079^T^ was distinct from other *Porphyrobacter* strains (Fig. 6, Additional file 4), which indicated that the amino acid frequencies of *P. cryptus* DSM 12079^T^ were also different from other strains with PC1 and PC2 accounting for 43 and 40% of the total variances, respectively. It was observed that the frequencies of asparagine, aspartic acid, glutamine, methionine, serine, threonine, tyrosine and valine were the lowest in *P. cryptus* DSM 12079^T^ proteins, whereas the frequencies of alanine, arginine, leucine and proline were the highest in this strain (Fig. 7). The *t*-tests performed on the frequency of these amino acids showed that the *p* values were all lower than 1.0 × 10^− 4^, indicating that these differences were significant.

The results showed that the major amino acids with net gains were alanine, arginine, glutamic acid, isoleucine and serine based on the substituted/substituting residues and amino acid frequency analyses (Figs. 5 and 7). Substitutions of these five amino acids occurred in 1039 proteins out of all 1405 shared and orthologous proteins in the genus *Porphyrobacter*.

Except for 12 overly long proteins that failed to be analyzed in secondary structure predictions, the percentages of α-helices, β-sheets, random coils and turns were identical in the 291 original and substituted protein pairs. However, the percentage of α-helices were clearly lower when the substitutional proteins were compared with their corresponding original proteins in *P. cryptus* DSM 12079^T^ (Additional file 5). Furthermore, 37 conserved proteins were selected through a comparative genomic study of selected *Alphaproteobacteria* genomic data. Except for five proteins not showing amino acid substitutions, the percentage of α-helices decreased in 18 proteins (Additional file 6). Previous studies showed that net increases in α-helix content could enhance the thermal stability of proteins [36, 37]. It is speculated that a net increase in α-helix content plays a key role in raising protein thermostability in the genus *Porphyrobacter*.

**Fig. 8:**
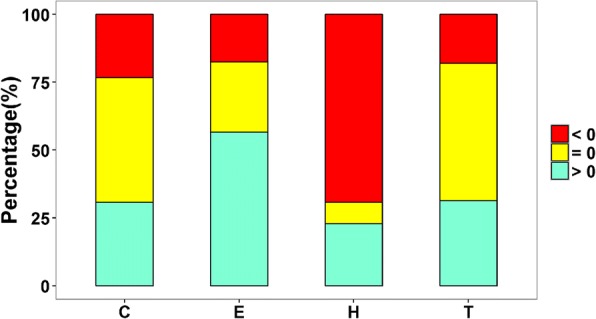
Variational percentages of protein secondary structures of *Porphyrobacter cryptus* DSM 12079^T^ original proteins to substituting proteins, according to sites where alanine was replaced by other amino acids. C, E, H and T stand for random coil, β-sheet, α-helix and turn, respectively. Red and green indicate decreases or increases in this kind of secondary structure, respectively, and yellow shows no changes

Further analysis of substitutional proteins with decreased ratios of α-helix regions indicated that 68.3% (348/509), 41.1% (209/509) and 39.8% (203/509) of these proteins had increased percentages of β-sheets, random coils and turns, respectively (Fig. 8**)**. It was found that alanine was the most commonly substituted amino acid (43.9%, 739/1682) among the five major substituted amino acids in substitutional proteins, resulting in fewer amino acids forming α-helix, and the most common replacement amino acids were glycine (20.8%, 154/739) and serine (21.4%, 158/739). In addition, *P. cryptus* DSM 12079^T^ had a distinctly higher frequency (*p* = 4.75 × 10^− 6^) of alanine than the other *Porphyrobacter* species (Fig. 7). Comparisons of secondary structures of *P. cryptus* DSM 12079^T^ proteins in which alanine was replaced by glycine/serine indicated that variations of secondary structures occurred in 57.1% (88/154) and 42.4% (67/158) of alanine to glycine/serine sites, respectively. It was investigated whether α-helices significantly changed into other secondary structures, and secondary structure changes occurred in 89.8% (79/88) of alanine to glycine sites and 94.0% (63/67) of alanine to serine sites. Previous studies showed that alanine was the best helix-forming amino acid [27] and that it was found at a higher frequency in thermophiles than in other organisms [38]. The side chain of alanine is methyl (-CH_3_), which promotes the interactions of neighboring residues in proteins and contributes to packing in protein structures [39]. In addition, hydrophobic residues located in the protein interior enhance conformational stability in the inner part of proteins [31]. Glycine and serine are both hydrophilic amino acids, and they can decrease the core hydrophobicity of proteins. Moreover, glycine tends to increase molecular flexibility by weakening protein stability at higher temperatures [40]; and serine can be released from proteins at higher temperatures, which results in unstable protein structures [41, 42]. Hence, alanine substituting for glycine or serine might promote the formation of α-helices in proteins, which could increase protein thermostability within *P. cryptus* DSM 12079^T^.

The analysis of neighboring amino acids of the substituted alanine indicated that hydrophobic amino acids occurred mostly in the N-terminus (41.1%, 58/141) and C-terminus (42.6%, 60/141), and this occurrence was higher than for other amino acid types. Furthermore, alanine, isoleucine and leucine were frequently located in the N-terminus, whereas alanine, leucine and proline were always found in the C-terminus among the neighboring hydrophobic amino acids of the substituted alanine. In addition, it was also often found that arginine and glycine were located in neighboring N- and C-terminal ends.

## Conclusions

Comparative genomics analysis of the thermophile *Porphyrobacter cryptus* DSM 12079^T^ with seven non-thermophilic *Porphyrobacter* strains broadened our knowledge of bacterial thermophilic mechanisms, especially in the class *Alphaproteobacteria*. Phylogenomic analysis of the genus *Porphyrobacter* and alignments of proteins encoded by shared single-copy genes revealed that amino acid substitutions play an important role in the thermophily of *P. cryptus* DSM 12079^T^. As a whole, substitutions from glycine/serine to alanine in *P. cryptus* DSM 12079^T^ proteins increased the frequency of α-helices in these proteins, which could promote protein thermostability and ensure the growth of *P. cryptus* DSM 12079^T^ at high temperatures. It is hoped that the findings of this study will help in the design of thermostable proteins using techniques such as site-directed mutagenesis.

## Methods

### Range and optimum of growth temperature

Seven type strains of the *Porphyrobacter* species were purchased from Deutsche Sammlung von Mikroorganismen und Zellkulturen GmbH (*P. cryptus* DSM 12079^T^, *P. dokdonensis* DSM 17193^T^, *P. neustonensis* DSM 9434^T^ and *P. tepidarius* DSM 10594^T^) and Japan Collection of Microorganisms (*P. colympi* JCM 18338^T^ and *P. sanguineus* JCM 20691^T^). All type strains were cultured in appropriate media [2–6, 8, 9] and maintained in glycerol suspensions (30%, *v*/v) at − 80 °C. The temperature range for growth (4, 10, 15, 20, 25, 30, 35, 40, 45, 50, 55 and 60 °C) was inspected in the appropriate medium of each strain. The absorbance at a wavelength of 600 nm for *Porphyrobacter* cultures was measured using an ultraviolet and visible spectrophotometer (TU-1810, Persee).

### Whole-genomic sequencing, assembly and annotation

Except for the genomic sequences of *P. cryptus* DSM 12079^T^, *P. mercurialis* Coronado^T^ and *P. neustonensis* DSM 9434^T^ were acquired under NCBI GenBank accession numbers AUHC00000000, JTDN00000000 [43] and CP016033 [44], respectively. Genomic DNA of the five *Porphyrobacter* type strains was extracted according to the protocol of AxyPrep™ Bacterial Genomic DNA Miniprep Kit (Axygen®, Corning) and detected through 0.6% (*w*/*v*) agarose gel electrophoresis with λ-HindIII digest DNA (Takara, Dalian). Whole-genomic sequencing was carried out using Solexa paired-end sequencing technology (HiSeq2000 system, Illumina, USA) based on a whole-genome shotgun strategy in Anoroad Gene Technology Co. Ltd. (Beijing). Raw reads containing ≥9 bp of N were removed and trimmed by removing adaptor sequences. Clean reads were assembled using ABySS 1.9.0 [45]. The ORFs were predicted by Glimmer v.3.0 [46] based on assembled genomes and then annotated by using the online Rapid Annotation using Subsystems Technology (RAST) server [47]. Clusters of Orthologous Groups (COGs) protein annotations were carried out by the online WebMGA server [48, 49].

### Comparative-genomic and phylogenomic analyses

The predicted proteins of eight *Porphyrobacter* species as well as *Altererythrobacter epoxidivorans* CGMCC 1.7731^T^ (GenBank accession number: CP012669) were compared through OrthoMCL version 2.0 based on OrthoMCL algorithm [50, 51] to detect orthologous clusters (OCs) among the genomes of nine species. Pan- and core-genomic analyses were performed using in-house shell and R scripts **(**Additional file 7**)**. The BLASTp searches [52] of unique OCs harbored by each *Porphyrobacter* species against a nr database were used to detect the closest relatives with thresholds of the identity above 70% and coverage not less than the half-length of query annotated proteins. Moreover, conserved proteins were selected by comparative genomic analysis with other *Alphaproteobacteria* genomes (Additional file 8) using OrthoMCL version 2.0 [50, 51].

Single-copy shared OCs of eight *Porphyrobacter* species and *Altererythrobacter epoxidivorans* were screened by in-house shell scripts **(**Additional file 7**)**. Each single-copy shared OC was aligned by using MAFFT version 7 [53]. Aligned sequences refined by trimAL version 1.4.1 [54] were concatenated manually. Then, a phylogenetic tree was constructed by maximum-likelihood [55] algorithms with IQ-TREE 1.6.1 software [56] based on concatenated aligned single-copy OCs with bootstraps analysis set to 100 replicates, with the best amino acid substitutional model set as LG + F + R3 as proposed by IQ-TREE 1.6.1 software [56]. The maximum-likelihood phylogenetic tree was visualized using MEGA 7 software [57]. The t-tests of genomic size and the number of CDSs were calculated by using the MASS package within R programming language (https://cran.r-project.org/web/packages/MASS/index.html).

### Amino acid substitutional analyses

Single-copy shared OCs of eight *Porphyrobacter* strains were screened and aligned according to the methods described above. Each site of the aligned protein sequences was detected through MEGA7 software [57] to determine a mutational site, which was defined as cases where *P. cryptus* DSM 12079^T^ held a different amino acid and other *Porphyrobacter* species had another identical amino acid. The amino acid content of each *Porphyrobacter* species was summarized, and principal components analysis (PCA) was carried out by using the psych package within R programming language (https://cran.r-project.org/web/packages/psych/index.html). Proteins in *P. cryptus* DSM 12079^T^ involved in major types of amino acid substitutions with net positive variation were screened using R scripts. Substitutional proteins were created manually by replacing the substituting amino acids with the substituted ones. Original and substitutional proteins were submitted to Chou & Fasman Secondary Structure Prediction Server (http://www.biogem.org/tool/chou-fasman/) to predict their secondary structures based on the Chou & Fasman algorithm [58, 59]. Variations about the percentages of α-helices, β-sheets, random coils and turns in the original and substitutional proteins were summarized to check intrinsic mechanisms leading to the thermophily of *P. cryptus* DSM 12079^T^.

## Additional files

Additional file 1 Alignment results of protein amino acid sequences encoded by single-copy genes found in eight *Porphyrobacter* strains. (ZIP 1510 kb)

Additional file 2 Summary of site-directed amino acid variations of *Porphyrobacter cryptus* DSM 12079^T^. (XLSX 16 kb)

Additional file 3 Summary of the substituted/substituting amino acid in *Porphyrobacter cryptus* DSM 12079^T^. (XLSX 8 kb)

Additional file 4 Frequency of each amino acid in *Porphyrobacter* type strains. (XLSX 10 kb)

Additional file 5 Summary of protein secondary structure variations in site-directed substitutions. (XLSX 99 kb)

Additional file 6 Summary of protein secondary structure variations in conserved proteins among *Alphaproteobacteria* bacteria. (XLSX 12 kb)

Additional file 7 In-house shell and R scripts for pan- and core-genomic analyses and screening single-copy shared OCs. (DOCX 13 kb)

Additional file 8 Summary of sources of *Alphaproteobacteria* strains applied in investigations of conserved proteins. (XLSX 10 kb)

## Abbreviations

CDSs: Coding sequences
NGS: Next-generation sequencing
OTG: Optimal temperature for growth
UTLG: Upper temperature limit for growth
