# Supplementary material for: Investigation of the thermophilic mechanism in the genus Porphyrobacter by comparative genomic analysis
Source: BMC Genomics. 2018 May 23;19:385. doi: 10.1186/s12864-018-4789-4 (PMC5966882; doi:10.1186/s12864-018-4789-4)
Supplement: Supplementary file 7 — In-house shell and R scripts for pan- and core-genomic analyses and screening single-copy shared OCs. (DOCX 13 kb) [file 12864_2018_4789_MOESM7_ESM.docx]

In-house shell and R scripts which are used to perform pan- and core-genomic analysis are listed below.

Shell scripts recording data

#!/bin/bash

#pan-genomic analysis

for line in $ (cat COMBINATION)

do

grep "File:${line}" "ORTHOMCL" > groups_tmp.txt

wc -l groups_tmp.txt >> pan-genomic.txt

done

#core-genomic analysis

for line in $ (cat COMBINATION)

do

grep -v "File:${line}" "ORTHOMCL" > groups_tmp.txt

wc -l groups_tmp.txt >> pan-genomic.txt

done

R scripts drawing a pan-genomic diagram

#loading ggplot2 package

library(ggplot2)

#reading input file

input <- write.csv("pan.csv")

#model building

model <- lm(Counts ~ Amounts + I(Amounts^2), input)

xmin <- min(input$Amounts)

xmax <- max(input$Amounts)

predicted <- data.frame(Amounts=seq(xmin, xmax, length.out=100))

predicted$Counts <- predict(model, predicted)

#drawing the pan-genomic diagram

ggplot(input, aes(x=Amounts, y= Counts)) + geom_point(alpha = 0.3, size=10) + geom_line(data=predicted, size=3, colour="blue") + theme(axis.text.x=element_text(colour = "black", size=rel(4))) + theme(axis.text.y=element_text(colour="black", size=rel(4))) + theme(axis.title.x=element_text(colour = "black", size=48, face="bold")) + theme(axis.title.y=element_text(colour = "black", size=48, face="bold")) + labs(x="Genomes", y="Pan-Genome Size")
